# Analysis of the Mechanical Behavior of Tree-like Fractal Structures in SLM-Manufactured Components

**DOI:** 10.3390/ma18102215

**Published:** 2025-05-11

**Authors:** Anca Stanciu Birlescu, Cristian Vilau, Nicolae Balc

**Affiliations:** 1 Department of Manufacturing Engineering, Faculty of Industrial Engineering, Robotics and Production Management, Technical University of Cluj-Napoca, Memorandumului 28, 400114 Cluj-Napoca, Romania; anca.stanciu@tcm.utcluj.ro; 2 Department of Mechanical Engineering, Technical University of Cluj-Napoca, 400114 Cluj-Napoca, Romania; cristian.vilau@tcm.utcluj.ro

**Keywords:** selective laser melting, mechanical behavior, tree-like fractals

## Abstract

Tree-like fractals as internal structures are a novel alternative to conventional lattice structures for mechanical components produced via Selective Laser Melting (SLM). This study explores the mechanical behavior of tree-like fractals, targeting flexure tests on SLM test samples manufactured using two distinct fractal configurations. The main objective is to develop numerical models that can predict the effect of the branching angle on the stress-strain curves, for both fractal configurations, from experimental flexure tests. A polynomial regression model is proposed to predict mechanical response variations based on fractal geometry, and the prediction model provides acceptable errors, less than the natural variance of multiple experiments. Furthermore, the tree-like fractal samples showed an interesting behavior on the flexure test, where the fractals deformed uniformly and in a predictable pattern, enabling mechanical advantages in impact absorption applications.

## 1. Introduction

The development of complex internal structures, e.g., lattices, in mechanical components attracted significant attention across various engineering applications [1]. These structures show specific mechanical advantages, e.g., lightweight design, high strength, and energy absorption capabilities. In material mechanics and science, internal structures (deployed inside a mechanical component) such as lattices offer specific advantages, namely: (i) high strength (relative to the volume of material) [1,2], (ii) good impact and mechanical energy absorption [2,3,4,5,6,7,8], (iii) crashworthiness [2], and (iv) good thermal conductivity [9,10].

A fractal is a recurring (non-smooth) geometry where the structure is preserved regardless of scale, i.e., the structure looks the same locally as globally, meaning fractals are self-similar and have space-filling properties [11]. These properties make fractals an alternative to lattices. The difference between lattice and fractals is that lattice structure has a unit cell pattern that repeats in (usually) translational symmetry, whereas fractals have irregular, self-similar patterns with scaling symmetry.

One of the various fractal structures is the tree-like fractal, which resembles the structure of trees, where each branch splits into smaller branches, repeatedly presenting self-similarity. Consequently, at each level of magnification, the pattern of branching is the same.

Tree-like fractals represent a novel approach for creating lightweight parts with internal structures (similar to lattice structures) for Selective Laser Melting (SLM), which is a branch of Additive Manufacturing (AM). As the literature shows, tree-like fractals were used for support structures in AM [12], but there is no evidence of using this approach for internal structures for cavity infill. To study the mechanical properties of tree-like fractals for internal structures, one must first define the geometric properties, generate the CAD design, manufacture the sample parts, and then conduct laboratory tests. A computational model was developed in [13] to allow a detailed modelling approach for fractal generation and populating the cavities of CAD parts with fractals; the approach enables full control of all the geometric parameters of the tree-like fractal, assuming that the fractal geometry can be manufactured by SLM (e.g., the branch diameter is not smaller than 0.5 mm, and the overhang angles are smaller than 45°).

Furthermore, in previous work [14], the mechanical properties of tree-like fractals were investigated based on experimental tests for bending and compression. The test samples were modeled such that tree-like fractals were connecting two plates, i.e., the tree-like fractals were sandwiched between two thin plates. The authors modeled test samples with two fractal arrangements, defined as S and SJ, respectively. The test samples were manufactured using SLM and were tested using standard equipment for bending and compression. The correlation between the FEA simulations (achieved in ANSYS2019 R2 Academic) and the numerical experiments, for both fractal configurations S and SJ, led to intriguing results that suggested that the geometry of the tree-like fractals influences the Young’s modulus of the materials; this result is not novel, as studies show how lattices change mechanical behavior by varying modeling parameters [15,16].

It is fairly difficult to determine the mechanical properties of one tree-like fractal based on its geometric parameters and then to generalize the global behavior of multiple fractals used in a manufactured part. Despite ANSYS being incredibly accurate, the tree-like fractals are designed with very narrow struts (on the order of 0.5 mm), and any manufacturing perturbation (from the original CAD) may lead to significantly different outcomes in mechanical testing. In addition to accurately computing the mechanical properties of tree-like fractals, manual meshing may be required for ANSYS, which is a time-consuming process. However, the mechanical properties of multiple fractal structures spread on a given volume may be investigated by considering a simpler problem. The test samples with S and SJ fractal configurations (investigated in [14]) are viewed as a meta-material (where the structure affects mechanical properties independently of material properties) with three layers (two thin layers sandwiching the fractal structure, assumed to be a homogeneous layer). If the mechanical properties (e.g., Young’s modulus) of this (assumed) homogeneous layer depend on the fractal structures’ geometry, it should be possible to generate a numerical model that describes mechanical properties as a function of the geometric parameters of the fractals. Curve-fitting approaches using linear polynomial regression can be used to develop such numerical models [17,18].

If the hypothesis that the geometric parameters of the tree-like fractals and the mechanical properties have a causal effect is correct, a model that describes this connection can serve as a building block towards understanding the tree-like fractals’ mechanical behavior on SLM components.

As a pilot study, this work will focus on the flexure tests with the two fractal configurations S and SJ [14] and with various angle parameters for the fractal structures. The scope of this work is to derive a numerical model of strain-stress curves for flexure tests of SLM samples with predefined tree-like fractals; to simplify the model, only the angle between branches is varied [13], whereas the other geometric parameters are kept constant. The outcome of this research is to provide a foundation in understanding the tree-like fractals’ mechanical behavior for SLM components. The contributions with respect to the state of the art are:

1. Providing statistical numerical proof that the geometric parameters of the tree-like fractals have a causal effect on the mechanical properties of the test samples.
2. Deriving a numerical model that can predict the shape of the stress-strain curves in flexure tests, based on the geometric parameters of the tree-like fractal structures.
3. Comparing the novel tree-like fractal approach for internal structures with different lattices to determine possible engineering applications for tree-like fractals.

The paper is structured as follows: Section 2 presents a background in the state of the art; Section 3 shows the methodology of this study, starting from designing the test samples, manufacturing, testing, and generating the numerical model; Section 4 shows the results of this study; Section 5 provides a discussion; and Section 6 presents the conclusions of the work.

## 2. Background

### 2.1. Mechanical Properties

Determining the mechanical properties of internal structures is an essential step in designing mechanical components with these internal structures. Energy absorption and strength are the two main properties that are discussed extensively in scientific literature. The review paper [2] highlights the lattice’s effectiveness in applications that require impact resistance and crashworthiness (ability to protect against mechanical impacts). Other focused studies showed the energy absorption properties of internal structures designed with multilayer internal structures [8], honeycomb patterns [3], dodecahedron structures [7], bionic fractal hierarchical structures [4], and other bioinspired patterns [5,6]. Furthermore, sandwich materials also benefit from the internal structure infill to achieve various properties [19,20] that can lead to superior mechanical properties suitable for structural applications. It is worth noting that complex internal structures differ in principle from foam materials [21,22].

AM also facilitates the manufacturing of components with internal structures. A review of applications and properties for the lattice structures manufactured using various AM technologies is presented in [21]; the authors compared various lattice structures with foam structures, emphasizing the advantages of the lattice where the dynamic load is known. In [20], the authors investigated the mechanical properties of lattice structures manufactured with SLM.

Thermal conductivity is another property that was studied in scientific literature, where AM-manufactured lattice structures showed desired properties [9,10] for conducting heat and creating heat sinks. Moreover, a review paper [23] discussed the effect of AM on residual stress on components; the authors argue that there are three main causes that produce residual stress: the AM technique of layer deposition, the direction of layers, and the temperature gradient. This also suggests that FEA is fundamentally a hard problem in AM components, especially with complex internal structures.

### 2.2. Mechanical Properties Analysis

Sandwiched materials with internal structures can be viewed as metamaterials. These kinds of materials require different testing and modeling than the traditional stress intensity factors and testing protocols [15]. A key observation is that different parameters used in constructing the internal structures significantly affect the mechanical properties of the metamaterial [16]. In [24], this effect is demonstrated on the impact resistance versus different structures of honeycomb core sandwich panels. The out-of-plane bending behavior of kerf chiral fractal lattice metamaterials was studied in [25]; the results suggest that these metamaterials exhibit specific properties that can be controlled by adjusting the fractal scales. Failure analysis on components manufactured via SLM was also studied in [16,26].

Numerical models for stress-strain (σ-ε) curves can be achieved via curve fitting [17,18]. One of the simplest models is based on a polynomial curve fit of various degrees. However, if the polynomial curve fitting is employed, one must account for the existence of Runge’s phenomenon [27], which produces oscillating behavior when computing the residual graphs between the prediction model and the real data. In [28], the authors proposed a reconstruction method to accurately predict complex residual stress profiles, overcoming the limitations of the traditional polynomial methods and enhancing prediction accuracy with limited experimental data.

The mechanical properties of lattice structures based on structural and material parameters were studied in [29]; the authors achieved a model that simplifies the analysis of large-scale structures with numerous lattice cells, offering a practical approach for the design of such materials. Furthermore, in [30], the authors presented a model that predicts the anisotropic mechanical properties of lattice structures based on their density and internal architecture.

In another review paper [31], the authors not only emphasize the mechanical properties of lattice structures but also the importance of numerical simulations in analyzing these structures and suggest that future research should focus on enhancing simulation techniques, developing new materials, and establishing uniform standards to fully harness the potential of metal lattice structures in engineering applications.

### 2.3. Discussion on the Tree-like Fractals with Respect to the State of the Art

Despite that there is no literature on tree-like fractals on SLM-manufactured components (to the author’s best knowledge), the knowledge regarding lattice structures is useful, and it is not far-fetched to assume that tree-like fractals may exhibit similarities with lattice structures.

There is no available research in scientific literature that compares tree-like fractals to lattices for SLM (to the authors’ knowledge). One initial step is indeed to determine the relation of geometric parameters of fractal structures to the mechanical properties of such materials, and an even simpler question is to determine the relation of a single parameter to the mechanical properties of components designed with tree-like fractal structures. This study has the potential to place the use of such fractal structures in the context of material mechanics, which in turn can generate predictions for the applicability of these structures in engineering.

## 3. Methods

### 3.1. Tree-like Fractal Definition

**Figure 1:**
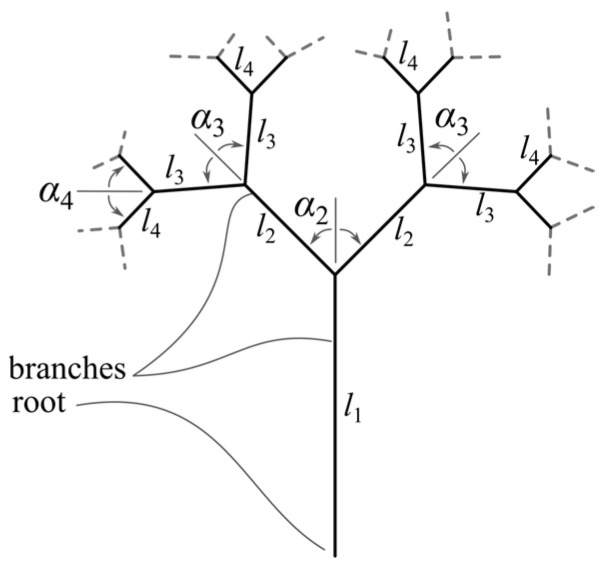
Representation of tree-like fractals and their geometric parameters.

In [13], the authors defined a tree-like fractal structure using only geometric concepts. Figure 1 shows a scheme of the proposed three-like fractals. The following parameters are defined (which allows the variation of tree-like fractal geometry):

- The branch pair length $l_{i},\;\;i=\bar{1,n}$ where *n* is the level (depth) of the fractal structure;
- The branch angle $\alpha_{i},\;\;i=\bar{1,n}$ that a child branch forms with its parent branch;
- The strut diameter d represents the diameter of a cylinder of height $l_{i}$.

The same mathematical apparatus [13] is used in this work; the tree-like fractals in this work are defined such that all angles between adjacent branches are equal (further referred to as *α*), and the lengths of the branches are $l_{0}=2.5\;\left[mm\right],\;\;l_{i}=l_{i-1}\cdot 0.8\;[mm]$ and the diameter of the struts is d=0.7 [mm]; in addition, a cutoff plane (for fractal trimming [13]) is defined at a height of 9 [mm] from the tree-like fractal’s basis (root). These design constraints ensure that the test samples (detailed later in the section) have the same dimensions (ensuring experimental consistency) and are dependent only on the angle parameter *α*.

### 3.2. CAD Modelling for the Test Samples

**Figure 2:**
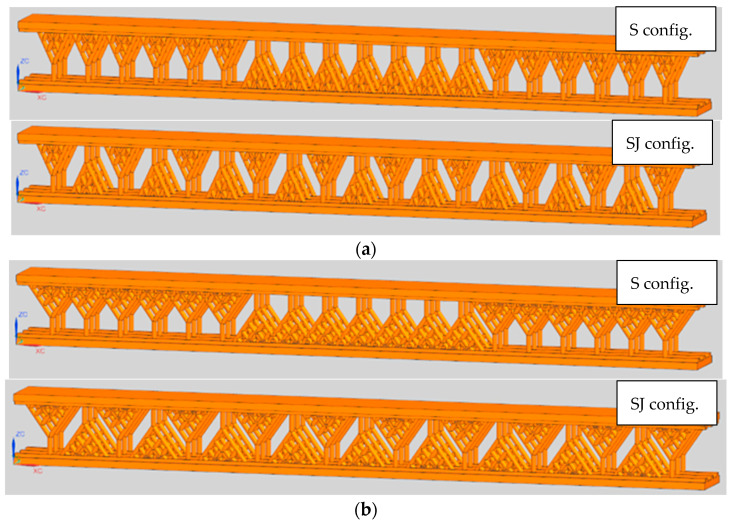
CAD models of samples with S and SJ tree-like fractal configurations: (**a**) *α =* 25°; (**b**) *α =* 35°.

Two configurations of tree-like fractals were defined, S and SJ, respectively, with different values of the angle parameter *α*. These configurations were used to design CAD models with α ∈ {25, 27, 30, 32, 35, 40, 45} [°]. The samples designed for α={25, 27, 30, 35, 40, 45} [°] are used to obtain data for the numerical regression (to derive the numerical model), whereas the samples designed for *α =* {32} [°] are for the validation of the regression model. Figure 2 shows the CAD models of both fractal configurations, S and SJ, using α ∈ {25, 35} [°]. Note that the samples were designed using the computational model developed in [13].

**Figure 3:**
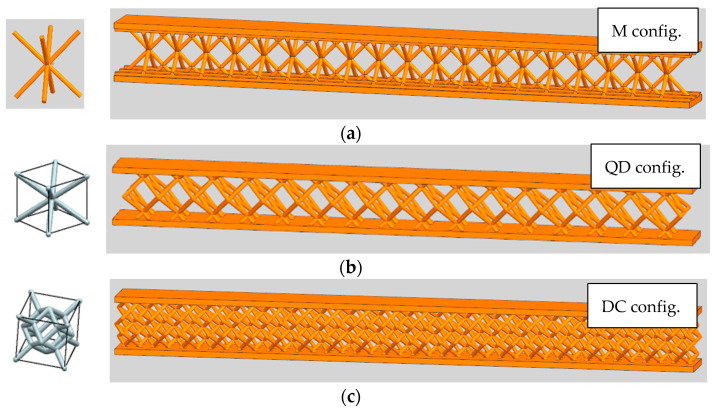
CAD models of samples with lattice structures: (**a**) M; (**b**) QD; (**c**) DC.

Furthermore, to compare the tree-like fractals with classical lattice structures, three configurations are defined for classic lattice configurations, namely M, QD, and DC (Figure 3).

The main dimensions were the same for all test samples (Figure 2 and Figure 3); namely, the length of the samples was *L* = 100 [mm], the height was *H* = 10 [mm], and the depth was D = 8 [mm]. In both S and SJ configurations, three fractal rows exist with a distance between the rows of 2 [mm]; the distance between the first branch of two fractals is 5 [mm] in all cases. In addition, the fractal layers were bounded by two thin plates, l_0_ = 1 [mm] plus 0.5 [mm] at the interface between the fractals and the thin plates (to accurately manufacture the treelike structures); the total number of tree-like fractal structures was 57 for both the S and SJ configurations.

### 3.3. Manufacturing the Test Samples

**Figure 4:**
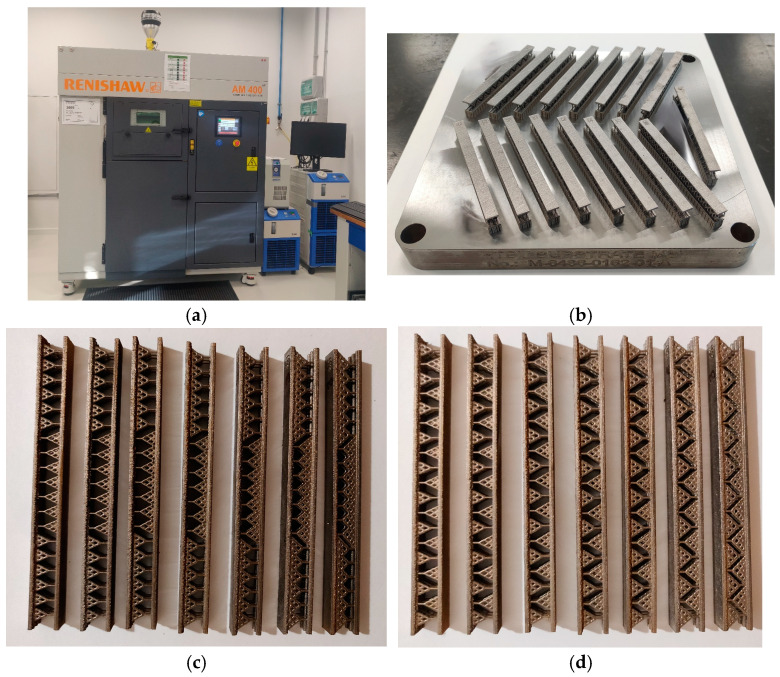
SLM manufactured samples: (**a**) the SLM equipment RENISHAW-AM400; (**b**) the manufactured test samples on the worktable; (**c**) the S configuration samples ordered ascending for α parameter (from left to right); (**d**) the SJ configuration samples ordered ascending for α parameter (from left to right).

The CAD models were manufactured using the SLM equipment RENISHAW-AM400 (Germany) using the material Tool Steel Powder 1.2709 from Böhler (Germany). Table 1 presents the printing parameters of the SLM equipment used specifically for manufacturing the test samples; Figure 4 shows the SLM equipment and the manufactured samples. Each test sample model was manufactured 3 times for consistency; however, only one batch of test samples is shown in Figure 4c,d.

There were no significant dimensional mismatches between the CAD models and the manufactured samples. All the measurements for the predefined lengths, *L*, *H*, *D*, and *l*_0,_ were within a defined tolerance of ±0.05 [mm]. Qualitative tests, such as rugosity and structural tests, are out of scope for this work.

### 3.4. Bending Experiments

**Figure 5:**
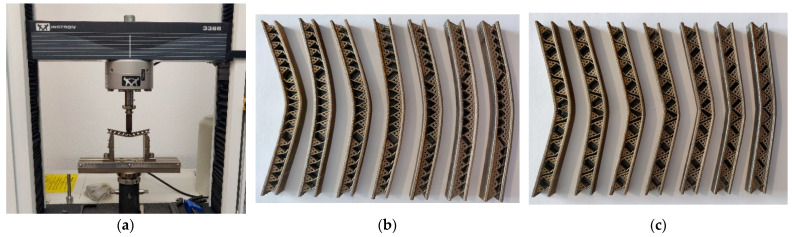
Experimental equipment and results for flexure tests: (**a**) experimental press Instron 3366; (**b**) S configuration samples after flexure tests; (**c**) SJ configuration samples after flexure tests.

The experimental samples were tested with bending tests using specialized equipment (Instron 3366 from Technical University of Cluj-Napoca, Figure 5). For each sample, the experiments recorded data until a threshold of σ = 7000 [Mpa] or until a fracture occurred on the sample. Since there were 3 samples for each model, the data were grouped accordingly to allow further processing (the baseline data were defined as the mean data between the 3 tested samples of each configuration/angle). The sampling frequency of the testing equipment was set to 10 [Hz]. Figure 5 shows the testing equipment with the mounted sample and the tested samples, deformed, after the bending tests. The testing equipment uses data denoising filters to cut off frequencies higher than half of the sampling frequency (the cutoff frequency was 5 Hz).

### 3.5. Testing the Cause-Effect Relationship

To determine if the geometric parameters of the tree-like fractal structures affect the mechanical properties of components, a simpler hypothesis can be formulated.

**Hypothesis 1.**  *The stress-strain curve σ-ε for bending tests of samples with the S, SJ tree-like fractal configurations depends on the angle parameter α; the relationship between the σ-ε curve and α is causal.*

To demonstrate the previous hypothesis, the stress is considered a function of both ε and α, σ=σ(ε,α). An experimental setup is designed to record data starting from the moment of initial contact between the sample and the tool, defined as t=0. The recorded data can only be defined in a discrete domain. A data vector ε is defined, together with a discrete function ${\left.f\right|}_{\varepsilon }$ that is used to compute a data vector $\sigma =[{\left.f\right|}_{\varepsilon }(\varepsilon ,\alpha )]$. Finally, the time vector t is defined with sampling time $t_{i}-t_{i-1}=0.1\;[s]$.

The causal relationship between the angle parameter α and the σ is considered true if both the following affirmations are true:

1. A correlation between α and σ exists, such that the shape of the σ-ε changes smoothly (not chaotically) with a change in α.
2. The trend produced by the correlation described at point 1 is reproduced for both S and SJ fractal configurations.

### 3.6. Defining the Numerical Model for the σ-ε Curve

In order to help the numerical regression in future steps, the following is considered.

**Assumption 1.**  *The first triplet of values* [t,ϵ,σ] *for time, stress, and strain, respectively, recorded at* t=0 *is* $\left[0,\;0,\;0\right]$*; consequently, the value of* $\sigma \in [{\left.f\right|}_{\epsilon }]$ *for* ε=0 *is* σ=0.

This allows for data normalization through $\epsilon =\epsilon -\varepsilon_{0}$ (which subtracts the first value of the vector ϵ from all the elements of the vector ϵ). Furthermore, the vector σ is normalized through $\sigma =\sigma -\sigma_{0}$. This normalization ensures that the data respects Assumption 1.

As previously stated, the set $\left\{25,\;27,\;30,\;32,\;35,\;40,\;45\right\}∋\alpha \;[{}^{\circ}]$ was used to manufacture all the test samples for both S and SJ tree-like fractal configurations. Two subsets are defined, $S_{M}\;:\left\{25,\;27,\;30,\;35,\;40,\;45\right\}∋\alpha \;[{}^{\circ}]$ being the subset used to generate data to develop the numerical model, and $S_{V}\;:\left\{32\right\}∋\alpha \;[{}^{\circ}]$ being the subset to generate data to validate the numerical model.

To determine a numerical model for predicting the σ-ε curves, the following regression models are defined:

1. A degree-9 polynomial model defined as

(1) $$poly9\;\equiv \sigma =\sum_{i=1}^{10}\left[p_{i}\cdot \varepsilon^{10-i}\right],$$

where the coefficients $p_{i},\;i=\bar{1,10}$ are assumed to be functions of the branch angles α; therefore, $p_{i}=p_{i}(\alpha )$.

2. A degree-3 polynomial model defined as

(2) $$poly3\;\equiv p_{i}=c_{i,1}\cdot \alpha^{3}+c_{i,2}\cdot \alpha^{2}{+c}_{i,3}\cdot \alpha +c_{i,4},$$

where the value of the angle parameter is restricted to $\alpha \in \left[25,\;45\right][{}^{\circ}]$.

In a nutshell, the regression model works in cascade: first, poly9 is used on the data sets $S_{M}\;:\left\{25,\;27,\;30,\;35,\;40,\;45\right\}∋\alpha \;[{}^{\circ}]$ to yield 6 sets of numerical values for $p_{i},\;\;i=\bar{1,10}$, and second, for each of the 6 values of $p_{i}$ the values of $c_{i,j},\;\;j=\bar{1,4}$ are computed. Furthermore, the poly9 model will be centered and normalized by the mean and standard deviation of ε, and the final model will have the general form of:

(3) $$Model\;\equiv \sigma =\sum_{i=1}^{10}\left[(c_{i,1}\cdot \alpha^{3}+c_{i,2}\cdot \alpha^{2}{+c}_{i,3}\cdot \alpha +c_{i,4}){\left(\frac{\varepsilon -\bar{\varepsilon }}{std(\varepsilon )}\right)}^{10-i}\right],$$

where $\bar{\varepsilon }$ and std(ε) are the mean value and the standard deviation of the data vector ε:[D].

**Figure 6:**
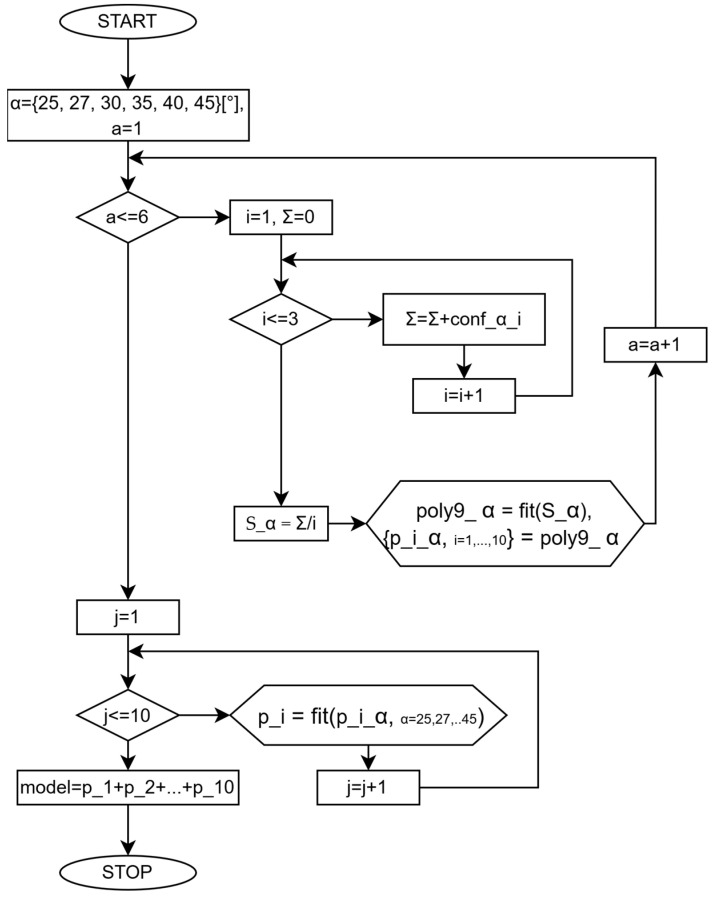
Flow chart representation for the numerical model.

Figure 6 shows a flow chart of the numerical model used independently for the S and SJ samples. The following are defined: fit() is defined as a fit function using regression; conf_α_i represents the data on a sample $i=\bar{1,3}$ (three specimens per experiment) with a configuration S or SJ, with angle α; poly9_α represents the fit model defined for each S_α sample; S_α represents the generic sample, which can be an S or SJ configuration with the angle parameter α; p_i_α represents the $p_{i}$ coefficients (Equation (1)) of every sample; p_i are the coefficients computed using the regression model defined in Equation (2); model represents the final model defined in Equation (1).

## 4. Results

The section is divided into two main sub-sections. On the one hand, the results leading to the development of the numerical model for σ-ε curves are presented. On the other hand, the results regarding the lattice-manufactured samples during the flexure test are shown (for comparison purposes).

### 4.1. Results on the Numerical Model for σ-ε Curves Prediction

**Figure 7:**
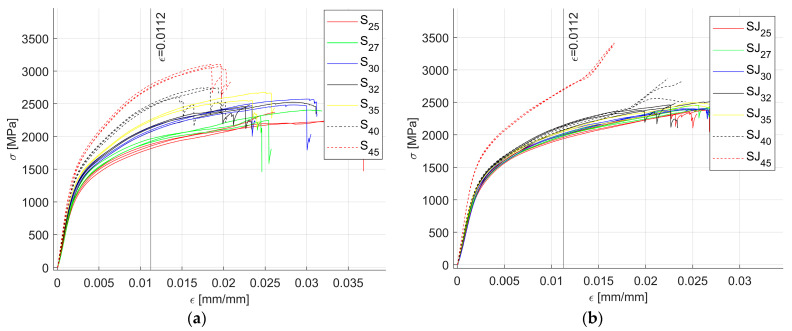
Raw data recorded for the flexure tests: (**a**) all S samples; (**b**) all SJ samples.

Figure 7 shows all the data recorded for all the test samples, highlighting the fracture instances as spikes in the curves. Since multiple fractures occurred at different ε values, a cut-off value for ε = 0.0112 (limiting the data to the first 600 sample points) was set to exclude any fracture since they have chaotic behavior, which cannot be modeled using the defined regression approach.

**Figure 8:**
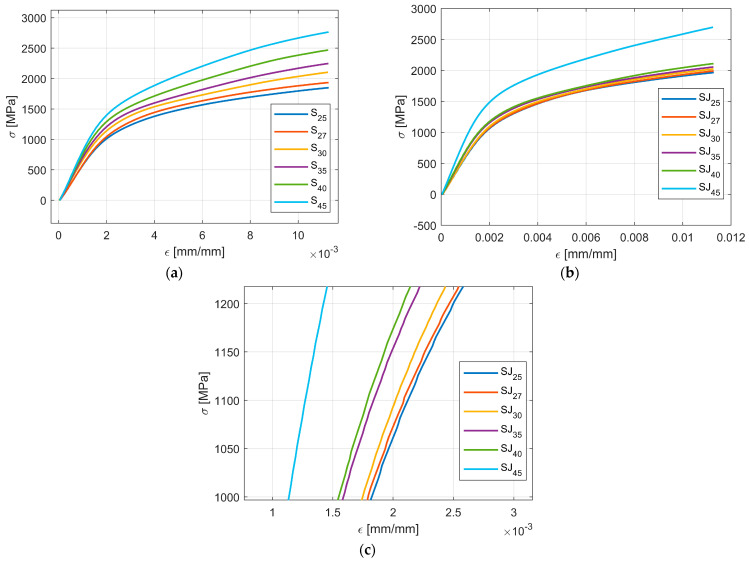
Processed data for the flexure tests: (**a**) all S samples; (**b**) all SJ samples; (**c**) zoom on SJ data.

Figure 8 shows the fractal data after the first data processing was applied, namely, taking the mean values for all three samples that describe the same tree-like fractal configuration associated with an angle α. In addition, the data are trimmed to 600 sample points. As stated before, the data sets used in computing the regression model were {25, 27, 30, 35, 40, 45} [°].

As seen in Figure 8, the change in the curve shape due to a change in the angle parameters appears smooth and ordered, which is the first clue that there is a causal relation between the values of α and the shape of σ-ε curves.

For each data set, $S_{k},\;k\in \{25,\;27,\;30,\;35,\;40,\;45\}$ a regression model was fitted using the fit() function within MATLAB 2024 using ‘poly9′ regression model (defined in Equation (1)) with the linear least squares algorithm, having the robust option set to ‘off’. Table 2 and Table 3 show the computed polynomial coefficients (of the S and SJ configurations, respectively) for the regression model, $p_{i},\;\;i=\bar{1,10}$; Table 4 and Table 5 show the statistical measures that interpret the goodness of the regression fit for every data set $S_{k}$ and ${SJ}_{k},\;\;k\in \{25,\;27,\;30,\;35,\;40,\;45\}$ respectively. Note that the presented numerical values are rounded, and the actual values used in the regression model are used with 15 decimal places. All regression models were normalized with the following mean and standard deviation:

(4) $$\bar{\varepsilon }=0.00561,\;std\left(\varepsilon \right)=0.00325,$$

**Figure 9:**
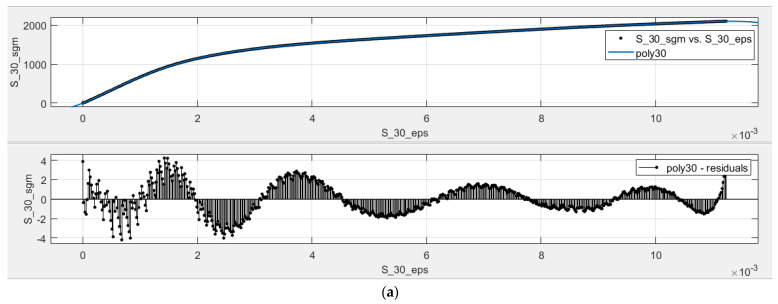

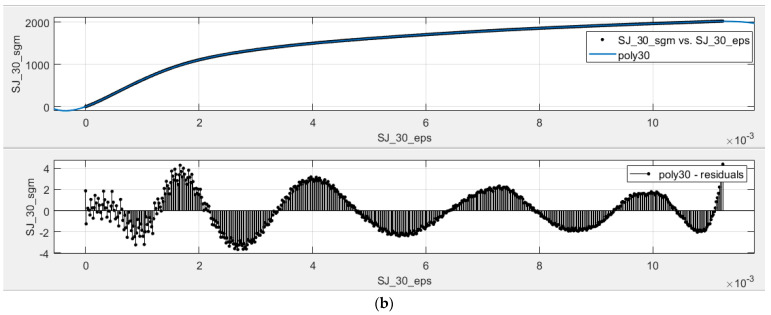
Fit curve and residual plots for α = 30 [°]: (**a**) S configuration; (**b**) SJ configuration.

Figure 9 shows the fit and the residuals for S and SJ fractal configurations. Figure 9a shows the fit between the poly9 (labeled as poly30) model and the S_30_ data (labeled as S_30_sgm vs. S_30_eps), together with the residual graph between the predicted values and the measured data (labeled as poly30-residuals), and Figure 9b shows the fit and the residual graph (labeled as poly30-residuals) of the poly9 model (labeled as poly30) of the SJ configuration and the SJ_30_ data (labeled as SJ_30_sgm vs. SJ_30_eps). There are two observations:

1. The polynomial model does not fit the data perfectly; the residuals show oscillating behavior, which implies that there is still some mathematical model that was not considered in the poly9 regression model. However, this oscillation may be a case of Runge’s phenomenon [27]. In addition, the oscillating behavior is insignificant with respect to the mean of the σ values; this effect accounts for 0.27% for S_30_ and 0.26% for SJ_30_, computed using $max(rezidual)/\bar{\sigma }$ , and this effect was lower than 0.31% for all data sets.
2. There is a sawtooth behavior on the residual graphs, which is most likely due to the micro-fractures on the sample during the laboratory experiments, resulting from the complex structures of the tree-like fractals, and secondly, from the way in which parts are manufactured using SLM.

In the second stage of regression, the aim was to compute a fit model, poly3, between the polynomial coefficients $p_{i,k},\;\;i=\bar{1,10},\;\;k\in \{25,\;27,\;30,\;35,\;40,45\}\;[{}^{\circ}]$. For each $p_{i},\;i=\bar{1,10}$ a regression model was fitted using the fit() function within MATLAB using ‘poly3′ regression model (defined in Equation (2)) with the linear least squares algorithm, having the robust option set to ‘off’. Table 6 shows the computed secondary coefficients $c_{j},\;\;j=\bar{1,4}\;$ for the S tree-like fractal configuration, whereas Table 7 shows the secondary coefficients for the SJ configuration.

The values of $c_{j},\;\;j=\bar{1,4}$ were used in the model shown in Equation (3) to create the final two models for the S and SJ configurations. It is expected that the computed model will not be as accurate as each regression model shown in Table 4 and Table 5. For each dataset $S_{k},{SJ}_{k},\;\;k\in \{25,\;27,\;30,\;32,\;35,\;40,\;45\}$ the fit quality was analyzed. Table 8 shows the statistical measures for the S configurations, and Table 9 shows the data for the SJ configurations. Note that the model predicts the validation data, namely the S_32_ and SJ_32_ data, respectively (grey highlight in Table 8 and Table 9). The normalized error (Norm Error) was computed using

(5) $$\frac{\left\|\sigma -Model\right\|}{\left\|\sigma -\bar{\sigma }\right\|},$$

where $\left\|\;\cdot \;\right\|$ is the Euclidean norm, σ is the data vector (*Y*-axis) recorded from the experiments, Model is the data computed with the prediction model, and $\bar{\sigma }$ is the mean of the recorded experimental data.

**Figure 10:**
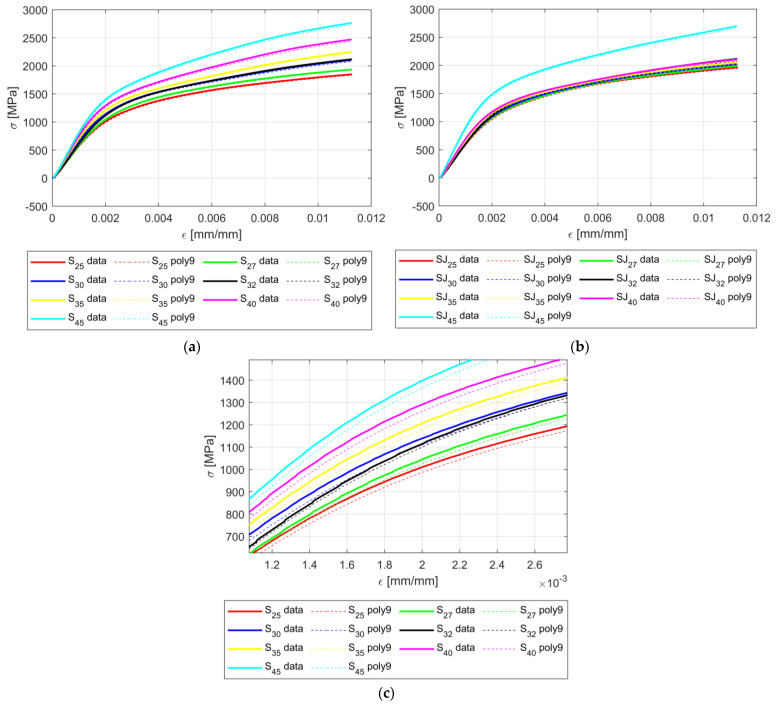
Comparison of the computed model and real data: (**a**) S configurations; (**b**) SJ configurations; (**c**) zoom on the SJ configurations.

As seen in Figure 10 the prediction model is underestimating the real data for all cases. In addition, the maximum error between the prediction model and the recorded data were 5.46%.

Figure 10 also shows two main behaviors of the σ-ε curves:

1. The angle parameter α indeed influences the shape of the σ-ε curves, and the behavior is consistent, gradual, and smooth; the influence of the angle parameter α is more pronounced for the S configuration (Figure 10a).
2. The tree-like fractal configuration influences the shape of the σ-ε curves; for the SJ configurations, the angle parameter α was not as pronounced (as for the S configuration) for the angle values {25, 27, 30, 32, 35, 40, 45} [°], i.e., the change in the aforementioned values was small. However, for the 45 [°] angle value, the change in the curve was abrupt.

The last step was to check the natural errors of each S and SJ specimen (three were manufactured for each S and SJ configuration associated with an angle α) to determine if the model is bounded between the natural variation of each set of experimental trials. Table 10 shows the errors calculated with respect to the mean values for each S fractal configuration associated with every angle α. Table 11 shows the errors for the SJ fractal configuration specimens. An orange highlight indicates the worst error results when compared with the numerical model prediction with respect to the mean. It can be noted that for the samples S_40_, SJ_35_, and SJ_45_ the errors for the prediction models with respect to the mean are worse than the natural error variation (with respect to the mean) of the three tested specimens. For all the other cases, the prediction model errors are lower than the natural errors.

### 4.2. Comparison of Lattice Structures and Tree-like Fractals Based on Flexure Tests

**Figure 11:**
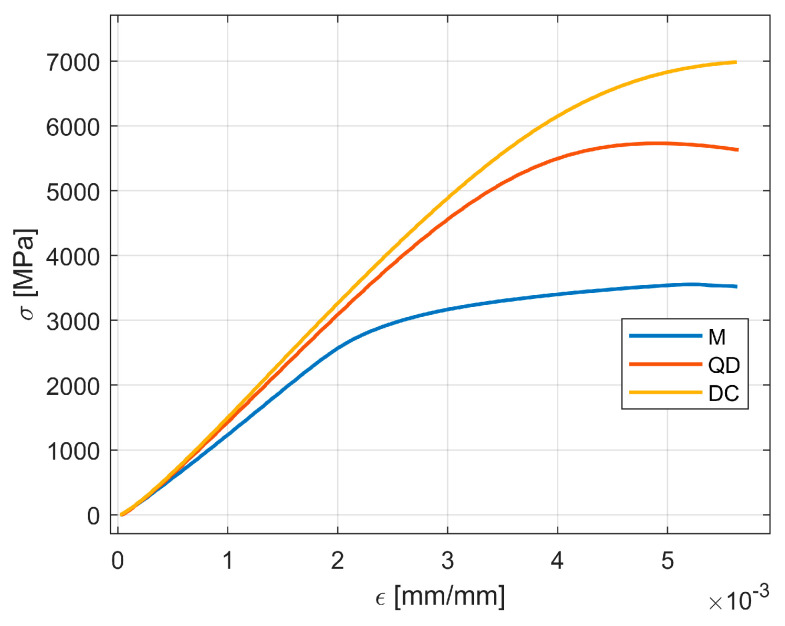
The lattice samples σ-ε curves on flexure tests.

**Figure 12:**
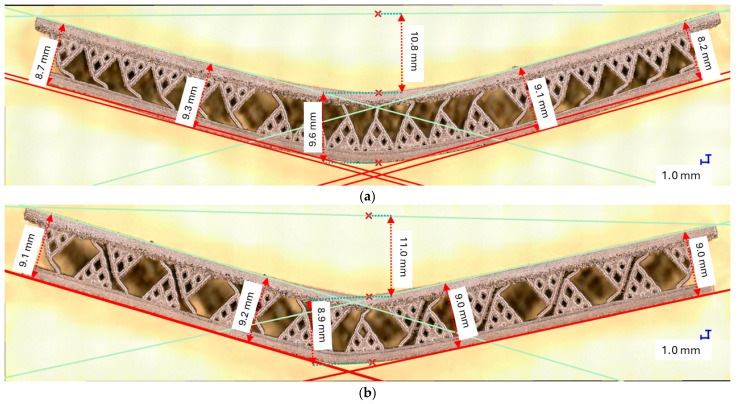
Optical microscope measures for the tree-like fractal structures: (**a**) S_30_; (**b**) SJ_30_.

**Figure 13:**
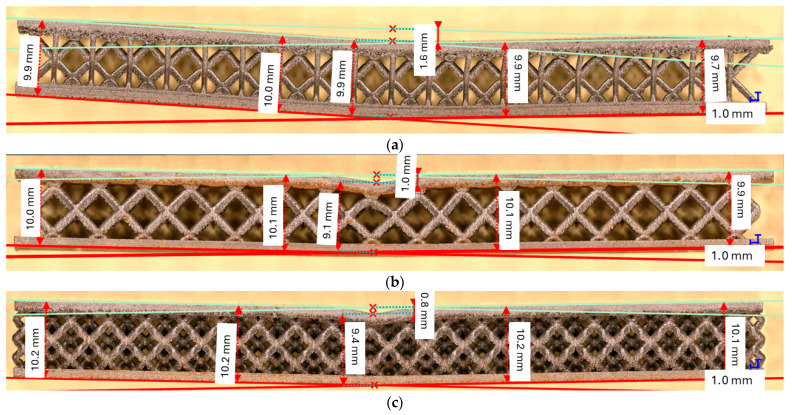
Optical microscope measures for the lattice structures: (**a**) M; (**b**) QD; (**c**) DC.

Figure 11 shows results for the samples manufactured with the defined lattice structures (M, QD, DC). All the lattice structures were manufactured in three specimens, and the data shown in Figure 11 is computed from the mean values of the specimens across σ and ε again up to a value of ε = 0.0056. It is easy to see that the lattices performed better when analyzing the component strength. However, an analysis under the optical microscope revealed an interesting behavior: the tree-like fractal samples deform in such a way that the thin layers that sandwich the fractal structures remain parallel to each other (Figure 12) up to a tolerance of a maximum of 0.9 [mm], showing a global deformation. However, the lattice samples (Figure 13) did not show the same deformation behavior, and the deformation of the thin layer appears local. Based on this result, a coefficient of flexure to compression can be calculated as

(6) $$C_{fl-c}=\frac{displacement\;of\;the\;thin\;plates\;}{distance\;between\;the\;thin\;plates},$$

and the results of this coefficient for each sample are shown in Table 12 for the S configuration samples (assuming the mean values of three tested specimens) and in Table 13 for the SJ configuration samples.

## 5. Discussion

### 5.1. Analysis on the Influence of Geometric Parameters on the Mechanical Properties of Tree-like Fractals

Figure 8 illustrates the shape of σ-ε curves based on the value of the angle parameter α. As seen in both cases, for the S fractal configuration and SJ fractal configuration, the trend remains the same, namely, an increase in the angle parameter α produces an increase in the σ to ε ratio, i.e., the apparent amplitude of the σ-ε curves increases with the increase in α. In addition, the change in the σ-ε curve shapes seems smooth for both fractal configurations S and SJ.

Based on the reported results, the following factual observations—(a) the change in the σ-ε curve shape is smooth in both fractal configurations (S and SJ); (b) the trend is the same in the σ-ε curve shape independent of the tree-like fractal configurations; and (c) the prediction models are smooth and yield errors that are bounded by the natural variance of each experiment—suggest strong evidence that the angle parameters α causally affect the shape of the σ-ε curves for the test samples designed with S and SJ fractal configurations. By extrapolation, we can infer that at least one geometric parameter (the angle parameter α) influences the mechanical behavior of the tree-like fractals.

### 5.2. Discussion on the Numerical Model for σ-ε Curve Prediction

Figure 9 shows oscillating behavior when analyzing the residuals between the poly9 model and the experimental data. On the one hand, this oscillating behavior suggests that there are additional considerations that were not accounted for in the poly9 model. On the other hand, these oscillations may be due to Runge’s phenomenon [27], and the oscillations are insignificant with respect to the mean of the σ values. The highest absolute values on the residuals (*y*-axis) with respect to the mean of σ were lower than 0.31% for all data sets.

Despite having a normalized error between 2.5% and 5.7% (depending on S or SJ configuration and angle parameter α), the initial statistical tests show that these errors are within the natural variation of the experimental trials. This suggests that the numerical models accurately describe the σ-ε curves based on angle values α∈[25, 45][°] for flexure experiments of samples designed with S and SJ fractal configurations. Note, if different fractal configurations are designed, the numerical models can be computed with the proposed methodology.

One intriguing result is that in all cases, the numerical model underestimates the mean value of σ-ε curves derived from experimental data. This effect can be corrected by multiplying the entire model with a factor (which may require numerical computation as it is most likely a function of α as well); however, since the numerical model yields good statistical results, such a factor may be irrelevant in practical applications.

It is worth pointing out that degree-9 polynomials were chosen simply because (for this study) they showed a good compromise between the complexity of the model (number of required coefficients) and the model error. Lower-degree polynomial models showed a rapid increase in error (with decreasing the degree), whereas higher-degree polynomials showed no significant improvement in the error while increasing in complexity.

### 5.3. Discussion on the Possible Applications of Tree-like Fractals

By comparing Figure 11 with Figure 8, it is obvious that lattices outperform the tree-like fractals when it comes to material strength by a ratio of about 3 to 1. However, this could be attributed to multiple design parameters; one possible explanation is that the tree-like configurations were designed to be planar in three layers, whereas the lattices were 3D (lattice structures were connected with all the neighboring structures).

Figure 12 shows a very interesting behavior where the tree-like fractal samples were deformed uniformly, and despite the action of the press being local, the deformation of the fractal configurations, both S and SJ, appears global. This is evidence of good impact absorption properties, but at this point, this property is just conjecture. Further (impact analysis) laboratory tests are required to evaluate the degree to which this property holds at high momentum impact. In addition, the flexure-to-compression coefficient should be investigated further as a continuous relation, since in the presented experiments, only two instances were measured (before and after the flexure tests).

In a nutshell, the results suggest that tree-like fractals (in the S and SJ configurations) underperform classic lattice structures in material strength but outperform them in impact absorption and controlled (predictable) deformability. Possible applications for tree-like fractals are materials designed to ensure safety (helmets, crush shields).

## 6. Conclusions

This research explored the mechanical behavior of tree-like fractals in Selective Laser Melting (SLM) manufactured components, focusing on flexure tests of two distinct fractal configurations (S and SJ). A numerical regression model was developed to predict the influence of the branching angle on stress-strain curves, demonstrating that geometric parameters play a crucial role in determining mechanical properties.

The results highlighted a causal relationship between the angle parameter α and the shape of the stress-strain curves, as the changes in α (branching angle) produced smooth and consistent variations in mechanical response. The proposed polynomial regression model achieved high accuracy, with errors generally within the natural experimental variance, validating its effectiveness in predicting mechanical behavior.

When compared to conventional lattice structures, the new tree-like fractals showed better deformability and impact absorption potential, even if the mechanical strength is lower than lattice structures. This suggests promising applications in safety-focused engineering designs, such as protective gear and energy-absorbing materials. Further studies, including impact resistance tests, are recommended to fully evaluate the practical advantages of these fractal structures.

By establishing a foundation for numerical modeling of tree-like fractals, this research paves the way for future advancements in lightweight, high-performance internal structures for additively manufactured components.

## Figures and Tables

**Table 1:** SLM printing parameters.

Power	Point Distance	Focus	Exposure Time	Scan Strategy	Layer Thickness
200 [W]	0.65 [µm]	5 [mm]	80 [µs]	11 Fill Hatch	0.04 [mm]

**Table 2:** Polynomial coefficients for the S configurations.

	S_25_	S_27_	S_30_	S_35_	S_40_	S_45_
*p*_1_	−8.766	−9.251	−7.849	−8.741	−13.58	−14.68
*p*_2_	13.15	12.61	11.01	13.84	19.66	19.75
*p*_3_	42.24	43.97	37.32	38.84	69.87	75.28
*p*_4_	−69.78	−61.13	−52.82	−69.56	−105.5	−106.9
*p*_5_	−35.21	−39.62	−28.14	−10.68	−72.31	−75.47
*p*_6_	59.38	29.55	4.115	24.73	88.85	91.93
*p*_7_	53.79	69.64	68.94	22.5	60.83	52.92
*p*_8_	−113.3	−94.34	−52.66	−44.41	−83.64	−108
*p*_9_	260.6	268.3	284.5	340	396.5	477.4
*p*_10_	1543	1609	1700	1784	1935	2155

**Table 3:** Polynomial coefficients for the SJ configurations.

	SJ_25_	SJ_27_	SJ_30_	SJ_35_	SJ_40_	SJ_45_
*p*_1_	−8.52	−8.937	−9.207	−10.91	−9.392	−14.49
*p*_2_	15.58	14.75	14.31	14.48	12.4	18.32
*p*_3_	40.43	43.74	41.97	55.84	47.33	75.18
*p*_4_	−85.5	−81.32	−71.96	−80	−66.35	−102.8
*p*_5_	−29.87	−36.29	−25.24	−53.98	−39.73	−72.83
*p*_6_	93.47	84.12	46.41	68.59	35.29	86.04
*p*_7_	49.19	50.57	42.53	58.04	53.16	66
*p*_8_	−154.7	−139.7	−100.3	−106.2	−69.19	−98.2
*p*_9_	287.6	293.7	297.5	284.4	292.8	382.6
*p*_10_	1651	1655	1672	1706	1723	2149

**Table 4:** Statistical measures illustrating the goodness of the regression fit (with respect to the means) for the S configurations.

Data Set	S_25_	S_27_	S_30_	S_35_	S_40_	S_45_
R-square	0.999	1	1	1	1	1
RMSE	1.533	1.954	1.548	1.021	1.614	1.842

**Table 5:** Statistical measures illustrating the goodness of the regression fit (with respect to the means) for the SJ configurations.

Data Set	SJ_25_	SJ_27_	SJ_30_	SJ_35_	SJ_40_	SJ_45_
R-square	1	1	1	1	1	1
RMSE	1.503	1.485	1.772	2.304	1.712	2.143

**Table 6:** Polynomial coefficients $c_{j},\;\;j=\bar{1,4}$ for every $p_{i},\;\;i=\bar{1,10}$ of the S configurations.

	*c*_1_	*c*_2_	*c*_3_	*c*_4_
*p*_1_	0.00369	−0.411	14.51	−172.6
*p*_2_	−0.00714	0.771	−26.68	310
*p*_3_	0.09617	−9.545	335.7	−2386
*p*_4_	0.04549	−4.936	171.9	−1995
*p*_5_	0.03205	−3.65	132.5	−1570
*p*_6_	0.09617	−9.545	335.7	−2386
*p*_7_	0.01023	−0.9635	28.79	−219.7
*p*_8_	0.04483	−5.329	204.1	−2585
*p*_9_	−0.00481	0.8331	−29.32	547.3
*p*_10_	0.09617	−9.545	335.7	−2386

**Table 7:** Polynomial coefficients $c_{j},\;\;j=\bar{1,4}$ for every $p_{i},\;\;i=\bar{1,10}$ of the SJ configurations.

	*c*_1_	*c*_2_	*c*_3_	*c*_4_
*p*_1_	−0.00373	0.3757	−12.52	128.1
*p*_2_	0.00438	−0.4252	13.37	−121.5
*p*_3_	0.01601	−1.595	52.92	−537.5
*p*_4_	−0.01901	1.773	−53.17	434.7
*p*_5_	−0.009319	0.8857	−28.97	288.7
*p*_6_	0.009873	−0.6363	6.898	160.7
*p*_7_	−0.002965	0.3645	−13.48	203.6
*p*_8_	−0.003835	0.04873	13.89	−469.5
*p*_9_	0.0697	−6.801	217.7	−1994
*p*_10_	0.2635	−25.41	808.5	−6807

**Table 8:** Statistical measures illustrating the goodness of the regression fit for the S configurations.

Data Set	S_25_	S_27_	S_30_	S_32_	S_35_	S_40_	S_45_
R-square	0.9985	0.9989	0.9978	0.9994	0.9984	0.9976	0.9980
RMSE	17.6982	16.0521	23.9920	13.1527	21.6658	29.0453	30.3138
Norm Error	0.0391	0.0335	0.0473	0.0252	0.0402	0.0486	0.0447

**Table 9:** Statistical measures illustrating the goodness of the regression fit for the SJ configurations.

Data Set	SJ_25_	SJ_27_	SJ_30_	SJ_32_	SJ_35_	SJ_40_	SJ_45_
R-square	0.9983	0.9993	0.9991	0.9970	0.9967	0.9991	0.9978
RMSE	20.1071	12.603	14.5798	28.5308	28.2720	15.024	29.6388
Norm Error	0.0416	0.0259	0.0293	0.0546	0.0576	0.0301	0.0470

**Table 10:** Statistical measures illustrating the errors of each for the S configurations when compared to the means.

Data Set	S_25_	S_27_	S_30_	S_32_	S_35_	S_40_	S_45_
R-square	0.9997	0.9962	0.9954	0.9923	0.9998	0.9991	0.9980
	0.9957	0.9984	0.9998	0.9967	0.9998	0.9998	0.9990
	0.9937	0.9986	0.9964	0.9989	0.9923	0.9987	0.9988
RMSE	7.4928	29.9404	34.9465	46.0927	7.5689	18.3042	30.6079
	30.2140	18.5991	7.1287	30.0516	7.6181	8.2983	21.4797
	35.6936	18.2014	30.0149	17.1135	25.5368	21.4758	22.5868
Norm Error	0.0167	0.0614	0.0676	0.0879	0.0139	0.0300	0.0451
	0.0659	0.0398	0.0142	0.0573	0.0143	0.0140	0.0310
	0.0794	0.0378	0.0598	0.0331	0.0503	0.0365	0.0340

**Table 11:** Statistical measures illustrating the errors of each for the SJ configurations when compared to the means.

Data Set	SJ_25_	SJ_27_	SJ_30_	SJ_32_	SJ_35_	SJ_40_	SJ_45_
R-square	0.9999	0.9992	0.9992	0.9923	0.9995	0.9999	0.9999
	0.9974	0.9996	0.9973	0.9967	0.9995	0.9981	0.9996
	0.9976	0.9999	0.9954	0.9989	0.9975	0.9980	0.9997
RMSE	5.5610	13.8868	13.9895	46.0927	11.3263	3.5869	7.4605
	24.9029	9.4921	25.7302	30.0516	11.2361	21.5234	12.1940
	23.4672	5.4167	34.7308	17.1135	24.4675	22.7371	10.6817
Norm Error	0.0116	0.0283	0.0288	0.0879	0.0232	0.0072	0.0119
	0.0507	0.0197	0.0517	0.0573	0.0228	0.0439	0.0193
	0.0488	0.0111	0.0681	0.0331	0.0501	0.0447	0.0169

**Table 12:** C_fl-c_ for the S configurations.

Sample	S_25_	S_27_	S_30_	S_32_	S_35_	S_40_	S_45_
C_fl-c_	1.6	1.3	1.2	1.2	1.0	0.6	0.6

**Table 13:** C_fl-c_ for the SJ configurations.

Sample	SJ_25_	SJ_27_	SJ_30_	SJ_32_	SJ_35_	SJ_40_	SJ_45_
C_fl-c_	1.1	1.2	1.2	1.2	1.1	0.9	0.6

## Data Availability

The original contributions presented in this study are included in the article. Further inquiries can be directed to the corresponding author.
